# The Usefulness of Reduced Anion Gap and the Possibility of Pupillary Findings in the Treatment of Acute Bromvalerylurea Intoxication

**DOI:** 10.7759/cureus.61781

**Published:** 2024-06-06

**Authors:** Saki Nakayama, Hideya Itagaki, Tomoyuki Endo

**Affiliations:** 1 Department of Emergency and Disaster Medicine, Tohoku Medical and Pharmaceutical University, Sendai, JPN; 2 Department of Emergency and Disaster Medicine, Tohoku Medical and Pharmaceutical University Hospital, Miyagi, JPN

**Keywords:** neurological pupil index, reduced anion gap, automatic pupillometer, bromism, bromvalerylurea

## Abstract

Bromvalerylurea is found as an over-the-counter analgesic and hypnotic drug in Japan and can be purchased at drugstores or over the Internet. Therefore, both acute poisoning due to large doses taken in suicide attempts and chronic poisoning due to continuous use for chronic pain have been observed. We report a case of acute BVU poisoning due to the use of an over-the-counter hypnotic sedative for a suicide attempt.

A 34-year-old woman was referred to our ICU with unexplained disturbance of consciousness, respiratory failure, and shock. During ICU management, when her pupil diameter was measured with an automatic pupillometer to confirm her conscious state, the right pupil diameter was larger than the left, but one hour later, the left pupil diameter was larger than the right. The difference between right and left fluctuated with the time of day. After awakening, it was found that the patient had taken 108 tablets of Utt, an over-the-counter hypnotic sedative, and the possibility of acute poisoning by its component, BVU, was raised. Because a blood gas analysis at the time of admission showed metabolic acidosis with anion gap ≤1, a diagnosis of acute BVU poisoning was made. The patient's general condition stabilized, and she was transferred to the psychiatric ward.

Symptoms of acute BVU poisoning include impaired consciousness and respiratory and circulatory depression, which may make it impossible to obtain a medical interview. When treating a patient with suspected drug intoxication who is unable to communicate, the clinician needs to include BVU poisoning in the differential when a reduced anion gap is observed. The clinician should also know that BVU poisoning can cause ocular manifestations such as anisocoria. This may lead to early diagnosis and therapeutic intervention.

## Introduction

Bromvalerylurea (BVU) is a bromide-containing monureide derivative with hypnotic and sedative properties, and originally it was used as a hypnotic drug [1]. It has been widely used as a hypnotic analgesic since the early 20th century, but its sale is now banned in most countries because of the risk of poisoning and suicide [2].

However, in Japan, BVU is an active ingredient in many over-the-counter (OTC) drugs. It is reported that 207 (29.1%) of the 711 OTC antipyretic analgesics sold in Japan contain BVU and can be purchased at drugstores or over the Internet without a prescription [2-4]. The OCT drug “Utt" is one such product, marketed as a hypnotic, containing 8 mg of diphenhydramine hydrochloride, 50 mg of allylisopropylacetylurea, and 80 mg of BVU a in one tablet. The blood half-life of BVU is short (2.5 hours), but the bromide ion, which is formed when BVU is metabolized, has a long blood half-life (12-14 days) and is excreted from the kidneys, making it easily accumulated in the body [5]. The toxicity of BVU is very strong, with a minimum intoxication dose of 4 g and a minimum lethal dose of 7 g. Therefore, both acute intoxication by large doses in suicide attempts and chronic intoxication by continuous use for chronic pain have been observed in Japan [1,4]．

In this study, we report a case of acute BVU poisoning in which 108 tablets of the OTC hypnotic sedative drug “Utt” (containing 83 mg of BVU per tablet) were taken in a suicide attempt, and anisocoria was observed, which changed over time.

## Case presentation

A 34-year-old woman presented to the emergency department with a disturbance of consciousness. Three months before her visit, she had been diagnosed by a psychiatrist with conversion disorder and was prescribed sleeping pills (Lemborexant). On the day of her visit, a colleague noticed that she did not show up for work and went to her home. She was found unconscious in bed and was transported to her family hospital. When transported to her family hospital, her vital signs at the time of transport were as follows: Glasgow Coma Scale of E1V1M1, blood pressure of 79/37 mmHg, pulse of 119 beats/min, body temperature of 38.5℃, respiratory rate of 23 breaths/min, and oxygen saturation of 80%. During the CT scan, the patient went into respiratory arrest and was intubated. Since blood tests and imaging studies showed no findings leading to impaired consciousness, drug intoxication was strongly suspected, and the patient was referred to our ICU. Vital signs at the time of transport to our hospital were as follows: Glasgow Coma Scale of E1V1M1, blood pressure of 82/52 mmHg, pulse of 91 beats/min, respiratory rate of 23 breaths/min, body temperature of 37.9℃, and oxygen saturation of 92% at fraction of inspiratory oxygen 50%.

Blood gas analysis showed a non-anion gap metabolic acidosis. Blood tests showed a mildly elevated inflammatory response and highly elevated CK (Tables 1-3).

**Table 1:** Complete blood count data and biochemistry data at the time of visit WBC, white blood cell; RBC, red blood cell; Hb, hemoglobin; Hct, hematocrit; Plat, platelet; MCV, mean corpuscular volume; MCH, mean corpuscular hemoglobin; Fib, fibrinogen; APTT, activated partial thromboplastin time; PT-INR, prothrombin time-international normalized ratio; TP, total protein; Alb, albumin; T-bil, total bilirubin; AST, aspartate aminotransferase; ALT, alanine aminotransferase; ALP, alkaline phosphatase; γGT, γ-glutamyl transpeptidase; LDH, lactate dehydrogenase; BUN, blood urea nitrogen; Cre, Creatinine; Na, Sodium; K, potassium; Cl, chlorine; Ca, calcium; Mg, magnesium; CK, creatine kinase; CK-MB, creatine kinase-myoglobin binding; Tropnin T, cardiac muscle troponin T; CRP, C-reactive protein; PCT, procalcitonin; IL-6, interleukin-6

Parameter	Result	Reference value
Complete blood count data		
WBC	10.7	3.3-8.6 (×10^3^/μL)
RBC	3.82	4.35-5.55 (×10^6^/μL)
Hb	12.4	13.7-16.8 (g/dL)
Hct	37	40.7-50.1 (%)
Plat	252	158-348 (×10^3^/μL)
MCV	96.9	83.6-98.2 (fL)
MCH	32.3	30.5-34.2 (pg)
Fib	226	200-400 (mg/dL)
APTT	33.1	24-39 (sec)
PT-INR	1.24	0.90-1.15
D-dimer	1.96	0-1.0 (μg/mL)
Biochemistry data		
TP	5.3	6.6-8.1 (g/dL)
Alb	3.6	4.1-5.1 (g/dL)
T-bil	0.59	0.4-1.5 (mg/dL)
AST	206	13-30 (U/L)
ALT	65	10-42 (U/L)
ALP	68	38-113 (U/L)
γGT	16	13-64 (U/L)
LDH	366	124-222 (U/L)
BUN	13	8-20 (mg/dL)
Cre	0.59	0.65-1.07 (mg/dL)
Na	136	138-145 (mmol/L)
K	4.1	3.6-4.8 (mmol/L)
Cl	105	101-108 (mmol/L)
Ca	7.8	8.8-10.1 (mg/dL)
Mg	1.8	1.6-2.6 (mg/dL)
CK	12,966	8-20 (U/L)
CK-MB	87	0-12 (U/L)
Tropnin T	<0.010	0.01-0.014 (ng/mL)
CRP	0.08	0.0-0.14 (mg/dL)
PCT	0.04	0.0-0.05 (ng/mL)
IL-6	771.8	0.0-7.0 pg/mL

**Table 2:** Blood gas analysis test at the time of visit BE, base excess; Lac, lactate; Na, Sodium; K, potassium; Cl, chlorine

Parameter	Result	Reference value
Blood gas analysis		
PH	7.332	7.35-7.450
pCO_2_	39.1	35-45 (mmHg)
pO_2_	123	80-100 (mmHg)
HCO_3_-	20.1	22-26 (mmol/L)
BE	-4.8	-2-2 (mmol/L)
Lac	1.2	0.4-1.8 (mmol/L)
Na	134	138-145 (mmol/L)
K	3.8	3.6-4.8 (mmol/L)
Cl	113	101-108 (mmol/L)

**Table 3:** Urine drug screen at the time of visit ー Indicates negative

Urine drug screen	
Methamphetamine	(ー)
Tetrahydrocannabinol	(ー)
Cocaine	(ー)
Benzodiazepine	(ー)
Barbiturate	(ー)
Tricyclic antidepressants	(ー)
Morphine	(ー)

**Figure 1:**
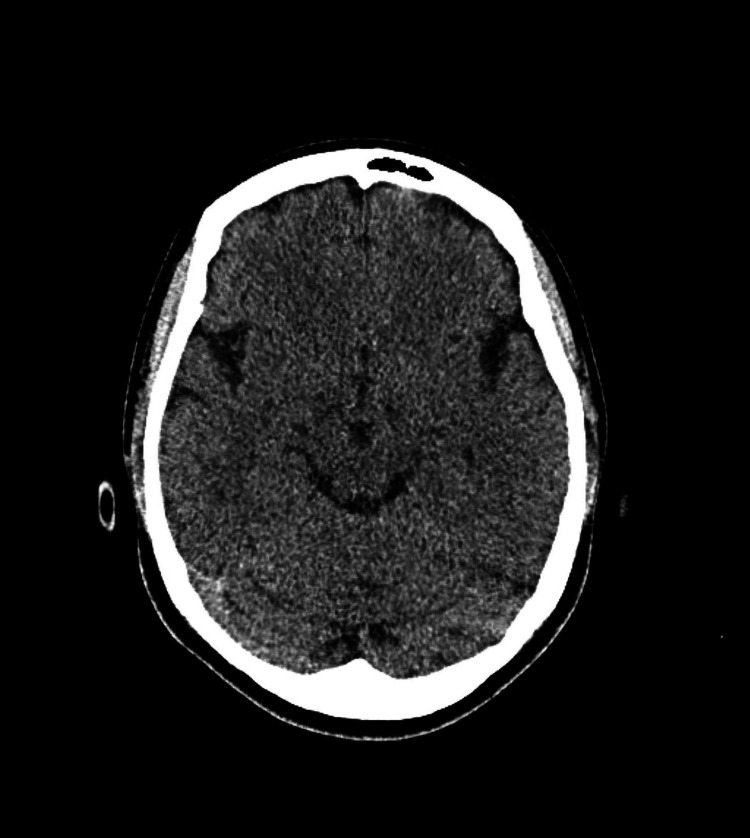
CT scan of the head showing no obvious abnormalities in the head

CT imaging showed no obvious abnormalities in the head (Figure 1), and consolidation was seen in the chest on both sides of the lungs, suggesting aspiration pneumonia.

**Figure 2:**
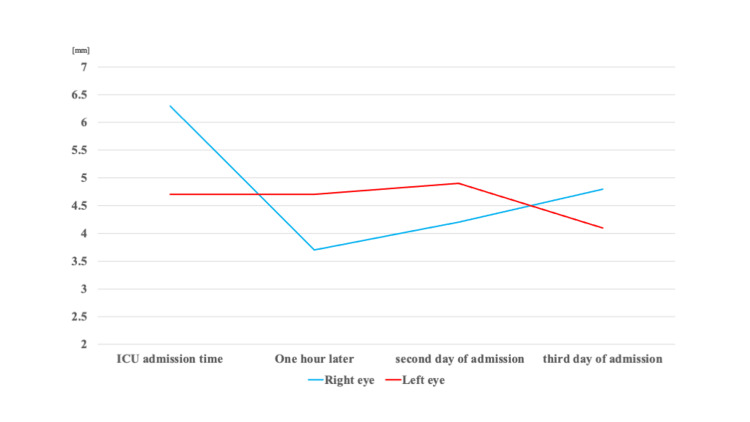
Time course of the pupil diameter

Blood pressure was hypotensive, and since rapid administration of extracellular fluid did not change the hypotension, we started a hypertensive drug, noradrenaline and vasopressin, and also administered 100 mg of hydrocortisone intravenously, and the blood pressure was kept stable. In addition, antibiotics (ampicillin sodium/sulbactam sodium) were administered for aspiration pneumonia. During the study, pupil diameter was measured quantitatively with an automatic pupillometer (Neuroptics NPi-200 pupillometer, NeuroOptics, Inc., Irvine, CA) to confirm the level of consciousness. Immediately after admission, pupil diameter was 6.3mm in the right eye and 4.7mm in the left eye, with the right eye larger than the left eye. One hour later, the pupil diameter was 3.7mm on the right and 4.7mm on the left, and the difference between the right and left was different at different times. Babinski sign was negative, and tendon reflexes were neither decreased nor increased, with no difference between right and left. However, on the second day of admission, the pupil diameter was 4.2mm/4.9mm, and on the third day of admission, it was 4.8mm/4.1mm, showing no more than 1.0mm difference between the left and right pupils (Figure 2).

**Figure 3:**
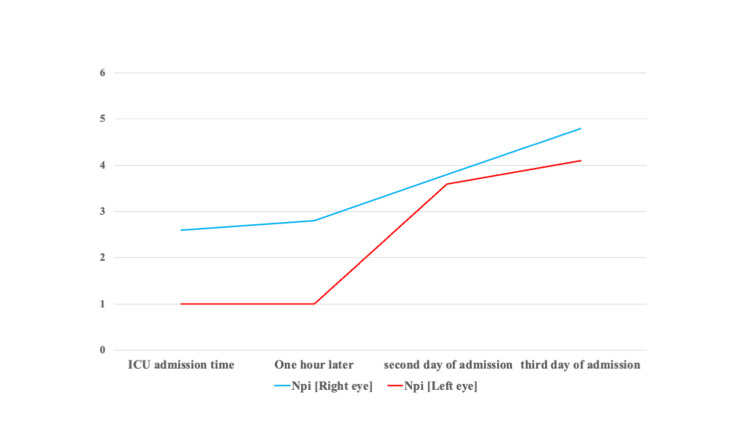
Time course of the neurological pupil index

The neurological pupil index (NPi) was also measured simultaneously. The NPi immediately after admission to the ICU and 1 hour later was 2.6-2.8 (right eye)/1.0 (left eye), but gradually improved to 3.8 (right eye)/3.6 (left eye) on the second day and 4.8 (right eye)/4.1 (left eye) on the third day of admission (Figure 3).

On the third day of hospitalization, the patient was able to discontinue the vasopressor and was weaned from the ventilator. After being weaned from the ventilator, she told me herself that she had taken 108 pills of the hypnotic sedative "Utt". The patient was suspected to have been poisoned by BVU, a component of "Utt", and blood gas analysis at the time of admission showed that the patient had metabolic acidosis with a reduced anion gap, which is seen in acute BVU poisoning. Therefore, the diagnosis of acute BVU poisoning was found to be consistent. Then, the patient was transferred to a psychiatric ward on the fifth day of admission to continue treatment for psychiatric disorders.

## Discussion

We reported a case of acute BVU intoxication with a reduced anion gap and an unequal pupil size. In addition, pupil size changed over time; initially, the right pupil diameter was larger, but, gradually, the left pupil diameter became larger. We learned the following lessons from this case. First, when a reduced anion gap is observed, bromism (BVU intoxication is also known as bromism) should be considered in the differential diagnosis, even if the serum chlorine is not elevated. Second, bromism can cause ocular symptoms such as anisocoria.

Chronic bromine poisoning is known to cause delirium, cerebellar ataxia, dystonic posture, dysarthria, psychiatric disorders, and cerebellar atrophy (especially in the cerebellar area of worms) [4, 6]. On the other hand, in acute bromine poisoning, bromide ions disrupt chlorine channels in extracellular fluid, muscle, and nerve tissue, resulting in impaired consciousness, respiratory and circulatory depression, liver dysfunction, and organ damage, such as gastrointestinal symptoms [3,5]. Treatment is based on discontinuing the causative drug and diuresis with saline solution, and dialysis is also effective [7]. Dialysis shortens the half-life of serum bromine to 1-2 hours, but even diuresis with saline solution alone can shorten the half-life to less than three days [7]. In our case, acute intoxication with BVU caused CNS depression, respiratory depression, and rhabdomyolysis. Dialysis was not introduced because the patient was treated with intravenous fluids, and urine output was maintained. However, the patient's symptoms were not diagnosed as being caused by BVU until she woke up. Bromism is diagnosed by measuring serum bromide ion concentrations, which is difficult to measure daily because it is unavailable at all facilities. Therefore, it is important to note that other laboratory findings in bromism include pseudo hyperchloremia and a reduced/negative anion gap [8]. Reduced anion gap means below the lower limit of normal. In bromide poisoning, the anion gap is usually less than 6 mEq/L (<6 mmol/L) [9]. Negative anion gap is an anion gap of less than 0 mEq/L [8]. It should be noted, however, that a reduced/negative anion gap can also be seen in hypoalbuminemia and hypercalcemia, hypermagnesemia, and hyperkalemia [8]. In the present case, the anion gap was ≤1, and since there was no hypoalbuminemia, hypercalcemia, hypermagnesemia, or hyperkalemia, it appeared to be a reduced anion gap due to acute bromism. If we focus on this reduced/negative anion gap, we may have been able to suspect acute bromism even before awakening. As a clinician, the lesson learned is to be aware of the reduced/negative anion gap when a patient presents with impaired consciousness.

In our experience, the above was the course of acute BVU poisoning, but in the present case, an atypical symptom of abnormal pupillary diameter response was observed. Although skin rashes called bromide rash and myoclonic jerks have been reported as characteristic physical findings of bromine poisoning, there have been very few reports on pupils [3,10,11]. Pupil size, reactivity, and symmetry changes in pupil diameter are used as indicators of worsening intracranial pathology, and the presence of intracranial lesions can be inferred by observing the size and reactivity of the pupil diameter [12]. However, the problem with penlight measurements is that they are often inaccurate due to high interrater variability [13]. For this reason, we use the Neuroptics NPi-200 pupillometer, an automatic pupillometer. This pupillometer also measures a composite value called NPi, which is calculated by a proprietary algorithm from the pupil size, latency of response, and rate of change of contraction/dilation, and the NPi is largely unaffected by pharmacologic effects [13]. If NPi is <3, the pupillary response is abnormal, and a lower NPi helps estimate the prognosis for intracranial hypertension and hypoxic encephalopathy [14,15]. Our patient had a left-right difference in pupil diameter, with an NPi of less than 3. We found only one case report of anisocoria caused by systemic administration of bromide. The case was anisocoria caused by systemic administration of ipratropium bromide, and it may not be strictly appropriate to treat it in the same way as BVU in the present case. However, Dax reported that 12 of 59 blood cases had ocular symptoms such as unequal or irregular pupils, nystagmus, and ptosis [16]. Although it is difficult to establish a clear relationship between anisocoria and bromine poisoning, the possibility that the anisocoria observed in this case was due to bromine poisoning cannot be ruled out in light of the aforementioned reports. The number of cases of BVU poisoning caused by OCT drugs in Japan is expected to continue. When drug intoxication is suspected in a patient with impaired consciousness and anisocoria is confirmed, the clinician may want to include bromine intoxication in the differential.

## Conclusions

We reported a case of acute BVU poisoning, causing impaired consciousness, respiratory and circulatory depression, and anisocoria. In this case, acute BVU poisoning was suspected for the first time during the post-awakening interview. Still, it is possible that acute BVU poisoning could have been suspected by focusing on the decreased anion gap from the beginning. In addition, ocular symptoms such as anisocoria may be seen in bromine poisoning. When anisocoria is seen in a patient with impaired consciousness, the physician needs to proceed with the differential with acute bromine poisoning in mind, in addition to the intracranial lesion.
